# Abortion

**Published:** 1868-12-15

**Authors:** 


### *EDITORIAL.*

***Abortion.***

"CINCINNATI, December 7.—In July last a young woman, named Georgiana Ridgeway, was killed by an abortion, performed by a quack, styling himself Dr. D. A. Ross. Ross was arrested, and this afternoon was sentenced to seven years in the penitentiary." The above is contained in the telegraphic summary of news in the daily papers. Its perusal suggests the fervent wish, that the same jury, which rendered a verdict apparently so righteous, might be induced to become a permanent attachment to the judicial system of this quack-ridden community. Scarcely a month passes during which the newspapers contain no record of "one more unfortunate gone to her death," through the diabolical practices of the abortionist. The suspected perpetrator is, possibly, arrested, but never punished, and never will be, until a radical change has taken place in public sentiment, until there shall arise an earnest desire to eradicate this crime, which shall, in its turn, suggest honest efforts to suppress it. It is this earnest desire which is just now wanting, and in making this assertion we do not feel that we are underrating the moral sense of the American people, which we believe to be as unerring in its indications as its enlightenment will permit; for we believe this want of desire proceeds solely from ignorance of the true character of this most prevalent crime. Impress this firmly upon the minds and hearts of the people, let them perceive the hideous reality, and the innate sense of rectitude of the human heart will effect what courts and juries now in vain attempt.

But how shall the moral sense of the public be enlightened, how shall they be compelled to see and know the truth? We answer, by keeping the *real truth* ever before their eyes; the truth naked, with all its deformities, uncovered by the drapery of deceptive and ambiguous phraseology, unvarnished by the gloss of social expediency and moral veniality.

But by what agency, and by what means, is this end to be accomplished? We believe that both are entirely within the scope of our own professional duties and capacities, and that the responsibility of the very existence of the crime, lies at the door of the medical profession. Let each physician, as a unit, satisfy his mind and conscience of his whole duty in this matter; and let him do it fearlessly, and *without respect of persons*, whenever occasion shall offer, and there will then be no necessity for co-operation, that tacit acknowledgment of integral weakness, which seeks support in mutual interdependence; no necessity for the profoundly moral preambles and denunciatory resolutions of Medical Associations and Social Science Congresses. We have said, that the responsibility of the existence of this crime lies at the door of the medical profession, for while a portion of it may, with apparent justice, be charged to the defects of our criminal code, which draws lines of demarkation between life and death, having no real existence, it must be remembered, that law, which, in the abstract, is but the expression of universal obligation and civil law, the expression in terms of civil and social obligation, is but a necessary deduction from science. If then the state of science be imperfect, its deductions must be erroneous. But science (that is the science of material things) is progressive, let law manifest progress also. It seems no more than simple justice, that a law, based in good faith, upon contemporaneous physiological knowledge, should be expunged from our statute books, now, when more comprehensive investigations into physiological phenomena have demonstrated the fallacious character of the foundation upon which it rests.

The civil law makes the production of abortion, before *the period of quickening* a *misdemeanor*, and subsequent to that period a *felony* — the punishment in each case being nominally apportioned to the offence. Now, the fœtus in utero is an organism, and, like every other organism, must be in one or the other of two categories, viz.: it must be either dead or living; if dead, it will, like every other dead organism, revert to the domination of chemical laws, and sooner or later be resolved into its original elements; if it then be not dead, it must be living, and potentially a human being, needing but a few weeks to develop its potential capacities into actual faculties. The law, therefore, which withholds its protection from this human being, which denies to it the privilege of life, because it is not yet capable of fulfilling all the functions pertaining to fully developed life, is as arbitrary and unjust as that which would refuse its protection to the life of citizen, because he had not yet reached the age assigned for the assumption of all the privileges of full citizenship. Is the youth of twenty years and six months less a citizen because he can not vote? And is the fœtus in utero less a human being because it can not yet breathe, or the infant of six months because it can not walk or talk? If it be so, where shall we fix the limit of life, where shall we place the threshhold of human existence, and by what physiological or other phenomenon is indicated this wonderful transition from death to life, from non-entity to entity? Where is the jurist who will answer? for physiologists dare not attempt it.

But this same civil law declares the malicious destruction of human life to be *murder*, and assigns as the punishment of murder, death. But the massacre of the innocents perpetrated daily, throughout the length and breadth of the land, is only a misdemeanor.

For the assassin, who for hire plunges his knife into the back of citizen, public sentiment demands the gallows, and the law awards it; but for the meaner, more cowardly assassin, who, for his thirty pieces of silver, plunges his probe into the brain of the citizens' unborn child, public sentiment is dumb, and justice is blind. But it would be unjust to medical jurisprudence, to ignore the unequivocal terms in which its highest authorities have characterized this crime, *at whatever period of intra-uterine life committed, as murder*. Here then we have the authority of prescript, were that necessary, to corroborate the deductions of right reason and sound philosophy, from correct physiological data. Let every physician do his whole duty in this work of enlightening the public mind upon this subject; let him not hesitate to protest against the commission of this most despicable form of assassination, under any and all circumstances, no matter what the exigency, and to lend his efforts to accomplish the punishment of its perpetrators with equal impartiality, and the crime will soon cease to be a possibility. Let him not be content to base his denunciations upon the unfavorable report of vital statisticians of the deleterious influence of fœticide upon populations in the gross, or upon individuals in detail, not upon the verdicts of Coroner's juries, as demonstrating its danger to material life, but upon that divine command, which has found its answering echo in the great heart of humanity, for six thousand years, "Thou shalt do no murder!" W. H.

